# The UK colorectal cancer screening pilot: results of the second round of screening in England

**DOI:** 10.1038/sj.bjc.6604089

**Published:** 2007-11-20

**Authors:** D Weller, D Coleman, R Robertson, P Butler, J Melia, C Campbell, R Parker, J Patnick, S Moss

**Affiliations:** 1 Community Health Sciences – General Practice, University of Edinburgh, 20 West Richmond Street, Edinburgh EH8 9DX, UK; 2 Cancer Screening Evaluation Unit, Institute of Cancer Research, Sir Richard Doll Building, Cotswold Road, Sutton, Surrey SM2 5NG, UK; 3 Bowel Cancer Screening Unit, Hospital of St Cross, Rugby CV22 5PX, UK; 4 NHS Cancer Screening Programmes, Don Valley House, Savile Street East, Sheffield S4 7UQ, UK

**Keywords:** colorectal neoplasms, mass screening, faecal occult blood

## Abstract

An evaluation of the second round of faecal occult blood (FOB) screening in the English site of the UK Colorectal Cancer Screening Pilot (comprising the Bowel Cancer Screening Pilot based in Rugby, general practices in four Primary Care Trusts, and their associated hospitals) was carried out. A total of 127 746 men and women aged 50–69 and registered in participating general practices were invited to participate. In all, 15.9% were new invitees not included in the previous round. A total of 52.1% of invitees returned a screening kit. Uptake varied with gender, age, and level of deprivation; was lower than in the first round (51.9 *vs* 58.5% *P*<0.0001), but was high (81.1%) in those who had participated in the first round with a negative result. Test positivity was 1.77%, significantly higher than in the first round, and the detection rate of neoplasia similar (5.67 per 1000), resulting in a lower positive predictive value. The sensitivity of FOBt in the first round was estimated as 57.7–64.4%. There was a significant impact on workload, particularly on endoscopy services. The cancer detection rate (0.94 per 1000) was lower than in the first round. Effort will be required to minimise inequalities in uptake, and to ensure adequate capacity of endoscopy services.

Colorectal cancer is a significant public health burden in the UK, and remains the most common internal malignancy (Wild *et al*, 2006). Randomised controlled trials have demonstrated that colorectal cancer mortality can be reduced by screening using the faecal occult blood test (FOBt) (Towler *et al*, 1998). In the light of this, a Pilot was established in the UK in 2000 to examine the feasibility of population-based screening for colorectal cancer. An evaluation of the first round of this Pilot has been reported previously (UK Colorectal Cancer Screening Pilot Evaluation Team, 2003; UK Colorectal Cancer Screening Pilot Group, 2004), and a national programme of screening began in England in 2006 and is being rolled out over several years (NHS Cancer Screening Programmes website). This paper reports on an evaluation of the second round of the Pilot in England (Weller *et al*, 2006); it provides detailed estimates of key outcome measures, including uptake of FOBt and colonoscopy, test positivity and detection rates of neoplasia, and a further analysis of the workforce and health service impact of bowel cancer screening.

## MATERIALS AND METHODS

### Screening pilot

The first round of the Pilot was conducted at two sites: the West Midlands in England and Tayside, Grampian, and Fife in Scotland. This evaluation of the second round includes data from the English site only. Men and women, aged 50–69 years inclusive, registered at general practices in Coventry Teaching Primary Care Trust (PCT), North Warwickshire PCT, Rugby PCT, and South Warwickshire PCT were eligible; however, due to competing service priorities, South Warwickshire PCT withdrew from the Pilot shortly after the commencement of the second round (only people in two practices in the PCT were invited). The policy was to invite people who would become 50 years of age during the year, and so in both rounds, there are a number of people aged 49 years of age. People aged 70 years or older who were registered with general practices in the Pilot area were able to request a kit by contacting the screening unit – strategies for this age group being made aware of the Pilot included information materials in doctors' surgeries, and receiving information from a spouse or other household member.

The English Colorectal Cancer Screening Pilot was administered from the Bowel Cancer Screening Unit (the screening unit) at the Hospital of St Cross in Rugby, which sent out invitations with Hema Screen test kits, comprising a card with six spots. Kits were returned to the laboratory; after testing (Phase 1 of screening) they could be negative, weak-positive (one–four spots), strong-positive (five–six spots) or inadequate. If the result was negative, the person was informed and no further action taken. If the result was weak-positive or inadequate, the person was sent another kit (Phase 2). Test-negative individuals from Phase 2 were sent a further kit (Phase 3). All those who had either a strong positive result at Phase 1 or returned any positive test at either of the two later phases were deemed to have a positive FOBt outcome and referred.

People who were referred were offered an appointment at the screening unit with a screening nurse who provided information and answered their questions. Bookings for screening nurse appointments and any investigations required (the standard follow-up for a FOBt-positive result was colonoscopy at the nearest endoscopy unit) were also arranged at the screening unit.

Screening for the second round began on 10 February 2003 and the last invitations were sent out on 9 November 2004. It was intended that the second round would take place at an interval of 2 years after the first round; however, there was a delay of 5 months before the start of the second round, due to programming and management constraints – consequently the median time between invitations was 28 months.

### Analyses of data

Routine individual-based data were extracted from the Pilot site database in June 2005. Additional information on the Index of Multiple Deprivation (IMD) and on ethnicity were linked to individuals using postcodes (Census Dissemination Unit website; Indices of Deprivation, 2004 website). Data on bowel cancers in people included in the first and/or second rounds were obtained from West Midlands Cancer Intelligence Unit, to identify cancers occurring in the interval between screening rounds. Data from the first and second rounds were linked by matching on NHS number and month/year of birth, to categorise people in the second round according to their screening experience in the first round.

To enable a valid comparison to be made between the two rounds, analyses were conducted on restricted populations from both rounds, including only people in those GP practices who were included in both rounds. We also excluded people aged over 70, except for analyses looking at self-referrals, and people participating in a trial of an immunological test in the second round (*n*=5122). Logistic regression was used to investigate associations between the demographic and ethnic variables and measures of uptake and positivity. Multivariate analyses including all demographic factors have been used to produce odds ratios of estimated effects adjusted for all other factors.

The test sensitivity of FOBt screening in the first round was estimated using the proportional incidence method (Day, 1985). Interval cancers included were those diagnosed within 2 years of a negative FOBt outcome, and before the date of any subsequent invitation; underlying incidence was estimated using both incidence rates for England in 2001 and those for West Midlands for 1998–2000. Cancers of the anus and anal canal were excluded from both the interval cancers and incidence rates. More details of the methods are given elsewhere (UK Colorectal Cancer Screening Pilot Evaluation Team, 2003).

### Impact evaluation

Activity data were used to examine any changes in Pilot-generated workload between the two rounds of screening; workload data on pathology, colonoscopy, radiology and surgical activity data were obtained from the first and second round Pilot databases. These were related to the total (unrestricted) population invited, but excluding South Warwickshire. In addition, screening activity was compared with total activity data obtained from each hospital. These data were supplemented by semi-structured interviews, held between December 2004 and December 2005, with key staff (endoscopy unit managers, colonoscopists and surgeons, colorectal cancer nurse specialists, pathologists, pathology laboratory managers, hospital managers, and screening unit staff). A general thematic analysis was undertaken using an iterative approach with analysis beginning after the first interviews to allow emerging themes to be explored in subsequent interviews.

## RESULTS

There were 127 746 invitees in the restricted second round population; 15.9% of people were new invitees, of whom 81.0% were aged 49–51 years. The proportion of people below age 55 years was slightly lower in the second round than in the first (30.5 *vs* 33.0%). The distributions of IMD were similar in both rounds, but there were a slightly higher proportion of people in ethnic minorities in the second round.

### Uptake

#### FOB test uptake

Of the 127 746 people invited, 52.1% (66 541) (95% CI: 51.8–52.4) returned a screening kit. Excluding from the denominator those tests returned by the post office (*n*=2185) and people whose screening episode was closed for one of several reasons (recent colonoscopy, moved from area, under treatment for bowel problems, deceased) (*n*=3504), the response rate was 54.5% (66 541/122 057).

Uptake (Table 1), defined as the proportion of those invited who returned an adequate kit in response to the invitation, was 51.9% (66 264/127 746) (95% CI: 51.6–52.1). This was lower than first round uptake (58.5% *P*<0.0001); this was true across all categories of the demographic variables (gender, age and deprivation). If the categories described above are excluded from the denominators, the uptake was 54.3% (66 264/122 057) and 60.6% (76 152/125 648) in the second and first rounds respectively.

Rates of return of inadequate kits were low; only 277 (0.4%) of those responding to the invitation failed to return an adequate kit in Phase 1 of screening. Some participants didn't complete the screening process; of 3105 people with ‘weak-positive’ test results in Phase 1, 217 (7.0%) failed to complete the screening process (at either Phase 2 or 3 of screening).

Uptake was high (81.1%) in those who had participated in the first round with a negative result, whereas for those who did not respond in the first round it was only 13.1%. In those aged 49–51 years, who were invited for the first time in the second round, uptake was 44.5%, compared with 51.9% in the same age group in the first round.

Uptake was also low in new invitees at older ages, 41.5% (522/1257) in those aged 60 years and over compared with 62.1% in this age group in the first round. Although the numbers are fairly small, this is the group most comparable to those to be invited in the planned roll-out of the screening programme.

Uptake was significantly lower in men than in women (47.7 *vs* 56.1% *P*<0.0001), and increased with age, from 45.7% in those aged under 55 years to 58.5% in those aged 65–69 years. People aged over 70 years were not invited routinely in the second round, but were able to request a kit from the screening centre. However, only 348 people did so, of whom 323 returned an adequate kit. Of the 323, only 113 were aged between 70 and 71 and so would have been invited in the first round.

Uptake fell with increasing level of deprivation, from 61.2 to 37.2% in IMD quintiles 1–5 respectively (test for trend *P*<0.0001), and was lower in areas with a high proportion of people from the Indian subcontinent (40.4%) than in areas with a low proportion (54.0% *P*<0.000l). These associations remained significant in the multivariate analysis (Table 1).

#### Colonoscopy uptake

A total of 1171 people had an overall positive FOBt outcome, of whom 1074 (91.7%). attended for a nurse appointment and 1001 were recorded as having been referred for colonoscopy, of whom 970 attended. Uptake of colonoscopy using number of positive FOBt outcomes as the denominator was 82.8% (95% CI: 80.6–85.0) in the second round compared with 80.5% (95% CI: 78.3–82.8) in the first round, but the difference was not significant (*P* 0.16). However, some people may have attended for private colonoscopy, on which we did not have information.

### Positivity

The positive rate, defined as the rate of a positive FOBt outcome in those returning an adequate kit, was 1.77%; this was significantly higher than that of 1.59% in the first round (*P* 0.01). As observed in the first round, the positive rate was higher in men than in women, and increased with age. The positive rate increased significantly with increasing level of deprivation, and was highest in areas with a high proportion of people of Indian subcontinent origin. These effects were reduced but remained significant in the multivariate analysis.

### Detection rates and positive predictive value

The detection rate of cancer was 0.94 per 1000; this was significantly lower than in the first round (1.35 per 1000, *P* 0.02). The detection rate was higher in men (1.40) than women (0.53) and increased with increasing age. The detection rate of neoplasia (both cancers and adenomas) was 5.67 per 1000, which was slightly lower than the first round (6.17 per 1000).

The positive predictive value of a positive FOBt outcome for cancer was 5.3%, and for all neoplasia was 32.1%. The positive predictive value for both cancer and neoplasia was significantly lower than that for the first round (8.5 and 38.8, respectively), but the difference is restricted to women.

A summary of the screening outcomes for the first and second round is shown in Table 2.

### Interval cancers and sensitivity

There were 98 interval cancers occurring within 2 years of a negative screen in the first round. The sensitivity of FOBt in the first round was estimated as 57.7% (95% CI: 48.4–65.6) or 64.4% (95% CI: 56.6–71.1) according to whether England or West Midlands rates were used to calculate expected incidence in the absence of screening (Table 3).

This estimate of sensitivity is similar to that of 62.7% observed in the Nottingham trial (Moss *et al*, 1999). However, in the Pilot sensitivity was higher in men than in women and this difference is in the opposite direction to that observed in the Nottingham trial.

### Impact of screening on hospital services

Our assessment of impact of screening on diagnostic and treatment services was based on procedures directly attributable to Pilot activity and comparisons with overall activity. Workload data generated in the first and second rounds of the screening Pilot (excluding surveillance colonoscopies), for people aged 50–69 years and 60–69 years, are shown in Table 4. There was no decline in colonoscopy activity in the second round. Further, screening-associated activity in the two main hospitals associated with the Pilot increased overall workload by approximately 14 and 28% respectively which was similar to the first round (Weller *et al*, 2006). In the second round there were fewer surgical operations and fewer bowel resection specimens to be examined in pathology departments; there was also less demand for radiology services (principally double-contrast barium enema).

Qualitative data from semi-structured interviews revealed a number of consistent themes. Firstly, the impact was felt most acutely among staff in endoscopy units; managing and performing screening-generated surveillance colonoscopies in a timely manner while meeting the demand for diagnostic work (both Pilot and non-Pilot) was challenging.

Secondly, for pathology services, the additional work created by the screening Pilot had most impact in the already overstretched and understaffed laboratories, although pathology staff were able to accommodate the extra workload. Finally, personnel involved in the provision of surgical services were aware of screening patients increasing their workload and the costs involved in terms of increased waiting times for non-urgent patients and the provision of extra operating and staging services. Further details of health service impact appear in the full report of the Pilot second round evaluation (Weller *et al*, 2006).

## DISCUSSION

This analysis of the second round of the English Pilot has provided the opportunity of examining how screening could potentially operate beyond the prevalence round as the programme becomes established in the UK. The dynamics of ongoing/periodic screening are different to those of a one-off prevalence type screen.

A key finding was the lower uptake of screening in the second round. The reasons for this are unclear; recruitment strategies were similar in both rounds, although there was greater publicity when the Pilot was launched, and this may have raised awareness. It is a form of screening, which is potentially distasteful, and requires considerable effort on the part of invitees – this may affect on-going participation. Consideration will need to be given in the roll-out process to devising ways of maintaining interest and motivation in a population, which is asked to participate in this form of screening every 2 years. It is also worth noting that other forms of FOBt such as immunochemical tests are available and may be easier to use (Young *et al*, 2002): the potential of such tests to produce higher levels of uptake warrants further exploration. The findings also reinforce the need to devise strategies to address low uptake in the subgroups which we identified. It would appear that low levels of uptake persist beyond the first round of screening in more or less the same pattern, and this will be an important consideration in reducing health inequalities in colorectal cancer incidence and outcomes (Smith *et al*, 2006).

The low number of people over age 70 who requested a kit is not unexpected, but consideration will need to be given to information needs for this age group as the Bowel Cancer Screening Programme rolls out; in the elderly it is especially important to weight the potential for harm from screening against the likelihood of benefit, given shorter life expectancy and greater comorbidity (Ko and Sonnenberg, 2005).

We have compared the positive rates in the Pilot with those of the first two rounds of the Nottingham trial, restricting both populations by age and uptake at first round to be comparable (Weller *et al*, 2006). The positive rate in the first round of the Pilot was slightly higher than that in the Nottingham trial (1.61 *vs* 1.38%). In Nottingham, the rate fell to 0.84% in the second round; the higher than expected overall positive FOBt outcome rate in the second round of the Pilot (1.8%) is therefore a cause for some concern. Clearly, the FOBt positive rate is the main driver for the rates of colonoscopy, and this is one of the key workforce/capacity issues in FOBt screening.

There was a drop-off in cancers detected in the second round, which is not unexpected in an ‘incidence’ *versus* ‘prevalence’ round of screening. The detection rate for all neoplasia (both cancers and adenomas) remained stable. Increasing positive rates coupled with falling cancer detection rates inevitably means that the predictive value for cancer of a positive test result is lower than in the first round. Positive predictive value is one of the most important markers in screening programmes; high rates of false positives lead to large numbers of unnecessary investigations being undertaken. Ultimately, this has an effect on cost effectiveness of screening (Pignone, 2005), and it will be important to monitor closely trends in positive rates over time as the programme rolls out; we have demonstrated that rates can vary considerably.

It will be particularly important to ensure adequate capacity in endoscopy units as the screening programme rolls out over the next several years (Tappenden *et al*, 2007). The colonoscopy rate per total invited population was similar in both the first and second round as although the positivity rate increased in round 2, the uptake of screening was lower. Importantly, the anticipated fall in demand for initial screening colonoscopies in the second round did not materialise, and our qualitative data further emphasise the impact of screening in endoscopic units. Colonoscopy services are frequently struggling to meet demand for symptomatic referrals. The Bowel Cancer Screening Programme has decided to bring management of screening surveillance colonoscopies into the screening programme; while this will not reduce the number of colonoscopies required, it will reduce the administrative work in the endoscopy units and enable the impact of the surveillance workload to be more clearly determined. There is on-going uncertainty over optimal colonoscopy intervals for adenoma/polyp surveillance (Mathew *et al*, 2006) and there is a need for more evidence to achieve national consensus on this issue if screening-generated surveillance is to be well planned, and incorporated into existing services.

England, Scotland and Wales are among the first countries in the world to introduce national programmes for colorectal screening. Our results suggest that on-going effort will be required to minimise inequalities in uptake by targeting deprived and certain ethnic groups, and to ensure adequate capacity – particularly in the provision of endoscopy services. It will be important to monitor performance measures such as uptake and positivity in this ‘roll-out’ phase, as these will give the first indication of the likely success of the programmes.

## Figures and Tables

**Table 1:** Uptake of screening by demographic factors

	**Uptake**			
		**Responded**		
	**Number invited**	***n***	**%**	**Adjusted OR (95% CI)**
Total	127 746	66 264	51.9	
				
*Gender*				
Male	64 373	30 711	47.7	1
Female	63 373	35 553	56.1	1.42 (1.36–1.48)
				
*Gender – age (years)*				
Male: <55	20 016	8275	41.3	1
Male: 55–59	18 710	8772	46.9	1.23 (1.18–1.28)
Male: 60–64	14 566	7434	51.0	1.47 (1.41–1.54)
Male: 65–69	11 081	6230	56.2	1.82 (1.74–1.91)
Female: <55	18 967	9528	50.2	1
Female: 55–59	18 209	10 239	56.2	1.26 (1.20–1.31)
Female: 60–64	14 520	8705	60.0	1.49 (1.43–1.56)
Female: 65–69	11 677	7081	60.6	1.55 (1.48–1.63)
				
*Deprivation category (IMD)*				
1 least	19 159	11 718	61.2	1
2	29 266	16 923	57.8	0.86 (0.83–0.90)
3	31 883	17 210	54.0	0.74 (0.72–0.77)
4	26 114	12 436	47.6	0.60 (0.57–0.62)
5 most	20 595	7655	37.2	0.41 (0.39–0.43)
Not known	729	322	44.2	
				
% *Indian subcontinent*				
Quintiles 1–4 (low)	105 883	57 148	54.0	1
Quintile 5 (high)	19 899	8039	40.4	0.89 (0.86–0.93)
Not known	1964	1077	54.8	

CI=confidence interval; IMD=Index of Multiple Deprivation; OR=odds ratio.

**Table 2:** Screening outcomes in first and second rounds of screening

	**Adequate return**	**Positive FOBT**		**Cancer**		**Neoplasia**		**PPV of positive test (%) (95% CI)**	
	***n***	***n***	**% (95% CI)**	***n***	**Rate per 1000 (95% CI)**	***n***	**Rate per 1000 (95% CI)**	**Cancer**	**Neoplasia**
*Second round*									
Gender									
Male	30 711	665	2.17 (2.01, 2.33)	43	1.40 (1.01, 1.89)	249	8.11 (7.14, 9.18)	6.47 (4.72, 8.61)	37.4 (33.8, 41.2)
Female	35 553	506	1.42 (1.30, 1.55)	19	0.53 (0.32, 0.83)	127	3.57 (2.98, 4.25)	3.75 (2.28, 5.80)	25.1 (21.4, 29.1)
Age at entry (years)									
<60	36 814	538	1.46 (1.34, 1.59)	16	0.43 (0.25, 0.71)	136	3.69 (3.10, 4.37)	2.97 (1.71, 4.78)	25.3 (21.7, 29.2)
60+	29 450	633	2.15 (1.99, 2.32)	46	1.56 (1.14, 2.08)	240	8.15 (7.15, 9.24)	7.27 (5.37, 9.57)	37.9 (34.1, 41.8)
									
Total	66 264	1171	1.77 (1.67, 1.87)	62	0.94 (0.70, 1.17)	376	5.67 (5.12, 6.28)	5.29 (4.08, 6.74)	32.1 (29.4, 34.9)
First round	76 152	1211	1.59 (1.50, 1.68)	103	1.35 (1.12, 1.64)	470	6.17 (5.62, 6.74)	8.51 (7.15, 10.40)	38.8 (36.0, 41.5)

CI=confidence interval.

**Table 3:** Test sensitivity, interval cancers, and person-years of observation within the 2-year period following the first round, by gender and age at entry

				**England**		**West Midlands**	
	**Person-years**	**Observed interval cancers**	**Rate per 1000**	**Expected cancers**	**% detected by screen**	**Expected cancers**	**% detected by screen**
*Gender*							
Male	100 626	49	0.49	140.5	65.1	169.0	71.0
Female	116 925	49	0.42	91.0	46.2	106.1	53.4
							
*Age at entry (years)*							
<60	126 346	36	0.28	75.2	52.1	91.9	60.8
60+	91 205	62	0.68	156.2	60.3	183.2	66.2
							
Total	217 551	98	0.45	231.5	57.7	275.1	64.4

**Table 4:** Summary of Pilot workload figures for ages 50–69 years for first and second rounds

	**Population^a^**	**Initial screening colonoscopies**		**Biopsy or polyp specimens**		**Resections specimens**		**Operations**		**DCBEs**	
	***n***	***n***	**Rate per 100 000**	***n***	**Rate per 100 000**	***n***	**Rate per 100 000**	***n***	**Rate per 100 000**	***n***	**Rate per 100 000**
First round	124 586	1006	807	1150	923	93	75	96	77	61	49
Second round	124 477	1026	824	1271	1021	59	47	59	47	38	31

DCBE=double-contrast barium enema; PCT=Primary Care Trust.

a The ‘denominator’ populations for these data are similar to the underlying populations used in Table 1, 2 and 3, except that they include people who were randomised to a trial of an alternative immunological test, and exclude invitees from two practices in South Warwickshire PCT and people aged 49 at invitation.
